# MicroRNA-Related Cofilin Abnormality in Alzheimer's Disease

**DOI:** 10.1371/journal.pone.0015546

**Published:** 2010-12-16

**Authors:** Jiaqi Yao, Tom Hennessey, Alex Flynt, Eric Lai, M. Flint Beal, Michael T. Lin

**Affiliations:** 1 Department of Neurology, Weill-Cornell Medical College, New York, New York, United States of America; 2 Department of Developmental Biology, Memorial Sloan Kettering Cancer Center, New York, New York, United States of America; Biological Research Center of the Hungarian Academy of Sciences, Hungary

## Abstract

Rod-like structures composed of actin and the actin-binding protein cofilin are found in Alzheimer's disease (AD) patients. However, the mechanisms underlying formation of these structures and their pathological consequences are still largely unknown. We found that microRNAs 103 and 107 repress translation of cofilin, and that reduced levels of miR-103 or miR-107 are associated with elevated cofilin protein levels and formation of rod-like structures in a transgenic mouse model of AD. These results suggest that microRNAs may play an important role in cytoskeletal pathology in AD.

## Introduction

In addition to amyloid plaques and neurofibrillary tangles, Alzheimer's disease (AD) brains contain Hirano bodies [1], which are rod-like structures composed largely of actin and the actin-binding protein cofilin [2], as well as other aggregates of actin and cofilin [3]. Cofilin regulates actin turnover [4]–[6], and abnormalities of cofilin directly affect the structure, dynamics, and functions of the cytoskeleton [3], [7]. In primary neurons, ATP depletion, excitotoxicity, and amyloid-β peptide treatment induce formation of cofilin rods [3]. These rods destroy microtubule bundles in neurites and interfere with neuritic transport and synaptic structure and activity [7], [8]. A recent study suggested that abnormal cofilin aggregation may initiate tau neuropil threads [9]. Thus, these cofilin-actin inclusions may play an essential role in AD pathogenesis. However, the mechanisms underlying cofilin-actin rod formation are still largely unknown.

MicroRNAs (miRNAs) have recently been implicated in neurodegenerative diseases [10], including Parkinson's disease [11] and AD [12]–[14]. Mature miRNAs are short (21–22 nt) noncoding RNAs that bind the 3′ untranslated region (UTR) of mRNAs and mainly repress translation. A large number of miRNAs are expressed in brain and potentially regulate expression of many genes. Of note, miR-107 is decreased in postmortem AD human brain, and has been associated with an increase in expression of β-site amyloid precursor protein cleavage enzyme 1 (BACE1) [12], thus linking miRNAs with an important suspected pathway in AD pathogenesis.

We show for the first time that (1) miR-103 and miR-107 repress cofilin translation, (2) reduced levels of miR-103 or miR-107 increase cofilin protein levels, and (3) increased levels of active cofilin protein leads to the formation of cofilin rods. Importantly, we show also that miR-103 and miR-107 levels are decreased and cofilin protein levels increased in brains of a transgenic mouse model of AD.

## Results

### Cofilin protein level is significantly increased in APP transgenic mouse brains and neurons

To determine whether cofilin expression level contributes to formation of rod-like structures, we examined cofilin levels in brains and primary neurons from Tg19959 mice, which overexpress human APP carrying the KM670/671NL and V717F familial AD mutations. Tg19959 mice develop brain amyloid deposition and cognitive deficits at the age of 4 months.

**Figure 1:**
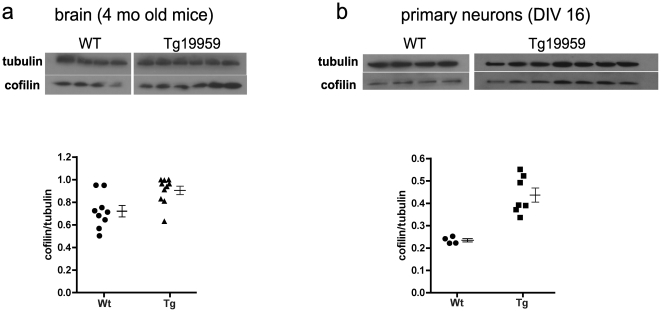
Cofilin protein level is elevated in APP transgenic mouse brain and primary neurons. (**a**) Western blots of brain lysates show elevated cofilin protein levels in 4-month-old Tg19959 mice compared to wildtype littermates. The graph plots the cofilin/tubulin ratios for the blots shown, together with data from an independent replicate (total n = 10 Tg, n = 9 Wt). Mean ± SEM is shown to the right of each group of raw data points. *P* = 0.0084 (two tailed *t*-test) comparing Tg (n = 10) vs Wt (n = 9). (**b**) Western blots of cell lysates show elevated cofilin protein levels in Tg19959 primary neurons (DIV 16) (n = 7 embryos) compared to wildtype littermate neurons (n = 4 embryos). The graph plots the cofilin/tubulin ratios for the blots shown. Mean ± SEM is shown to the right of each group of raw data points. *P* = 0.0012 (two tailed *t*-test) comparing Tg (n = 7) vs Wt (n = 4).

Protein extracted from 4-month-old Tg19959 mouse brains was analyzed by western blot. Brain levels of cofilin were increased 1.3-fold in Tg19959 mice compared to wildtype littermates (Fig. 1a). In cultured primary neurons (DIV 16), cofilin levels were increased 2-fold in neurons from Tg19959 embryos compared to those from wild-type littermates (Fig. 1b).

### Cofilin rod-like structures are detected in Tg19959 primary neurons and brain

**Figure 2:**
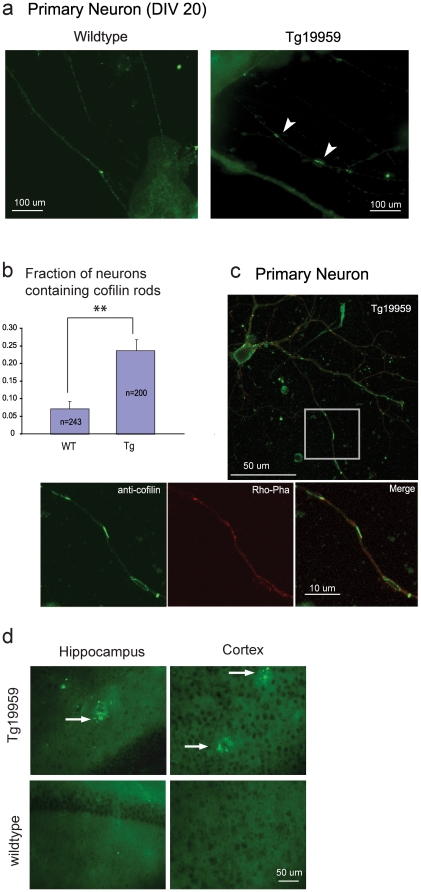
Cofilin rods are increased in APP transgenic mouse neurons compared to wild type littermate neurons. (**a**) Rod-like structures immunoreactive for cofilin (arrowheads) are detected in Tg19959 primary neurons (DIV 20) permeabilized with ice-cold methanol. Scale bars represent 100 µm. (**b**) The fraction of neurons (DIV 20) containing such rod-like structures is 23.9±3.1% in Tg19959 neurons (n = 200 neurons from 3 embryos), but only 7.3±2.1% in wildtype littermate neurons (n = 243 neurons from 3 embryos). **P = 0.00027, two tailed *t* test. (**c**) Rod-like structures immunoreactive for cofilin are detected in Tg19959 primary neurons permeabilized with Triton X-100. These structures are not stained by rhodamine-phalloidin. (**d**) Cofilin aggregates (arrows) are detected in 4 month old Tg19959 mouse brains but not in wildtype littermates. Scale bar represents 50 µm.

Cofilin and actin form rod-like structures in postmortem AD brains [1], [3]. When cells were permeabilized with ice-cold methanol (which is ideal for visualizing cofilin rods, [3], [15]), we also found rod-like structures immunoreactive for cofilin in Tg19959 primary neurons, but only rarely in wildtype littermate neurons (Fig. 2a). The fraction of neurons containing cofilin rods was 3-fold higher in Tg19959 neurons compared to wild-type neurons (23.9±3.1% vs 7.3±2.1%, Fig. 2b).

It has been previously described that cofilin-actin rods are not detected by rhodamine-phalloidin, even though rhodamine-phalloidin normally binds filamentous actin. It has been suggested that the actin cytoskeleton in this structure is saturated with cofilin protein, which blocks binding of rhodamine-phalloidin [3], [16]. As expected, when we permeabilized cells with Triton X-100 (ideal for visualizing actin, [17]), cofilin rods were not detected by rhodamine-phalloidin (Fig. 2c). However, the cofilin rods in Tg19959 neurons do contain actin, as shown by staining with a total actin antibody (Fig. S1).

In addition to primary neurons, we detected abnormal aggregates strongly immunoreactive for cofilin in cortex and hippocampus of 4 month old Tg19959 mice but not wildtype littermates (Fig. 2d). They are likely related to the pathologic process in AD because they are found in the vicinity of plaques.

### Cofilin mRNA levels are unchanged in APP transgenic mouse model compared to wildtype

**Figure 3:**
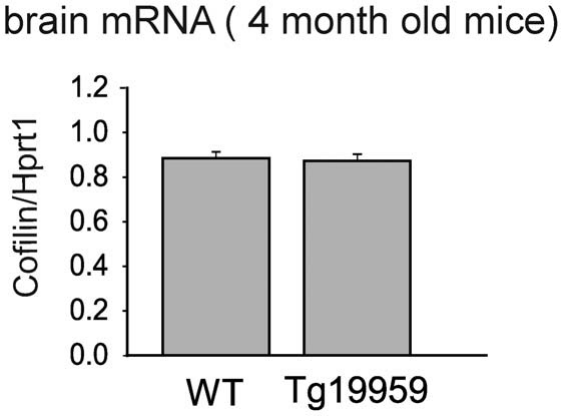
Cofilin mRNA levels are unchanged in APP transgenic mouse brains compared to wildtype brains. There is no change in cofilin mRNA levels, normalized to levels of hypoxanthine phosphoribosyl transferase (*Hprt1*) mRNA, in brains of Tg19959 mice (n = 8) compared to wildtype littermates (n = 9).

To investigate the mechanisms of increased cofilin protein levels, we first examined transcription. We extracted total RNA from brains of Tg19959 mice and wildtype littermates. Reverse transcription followed by real time PCR showed no change in cofilin mRNA levels in Tg19959 mice compared to wildtype littermates (Fig. 3, three independent trials). Therefore, the mechanism leading to increased cofilin protein levels occurs post-transcriptionally.

### MiR-103 and miR-107 repress cofilin translation

**Figure 4:**
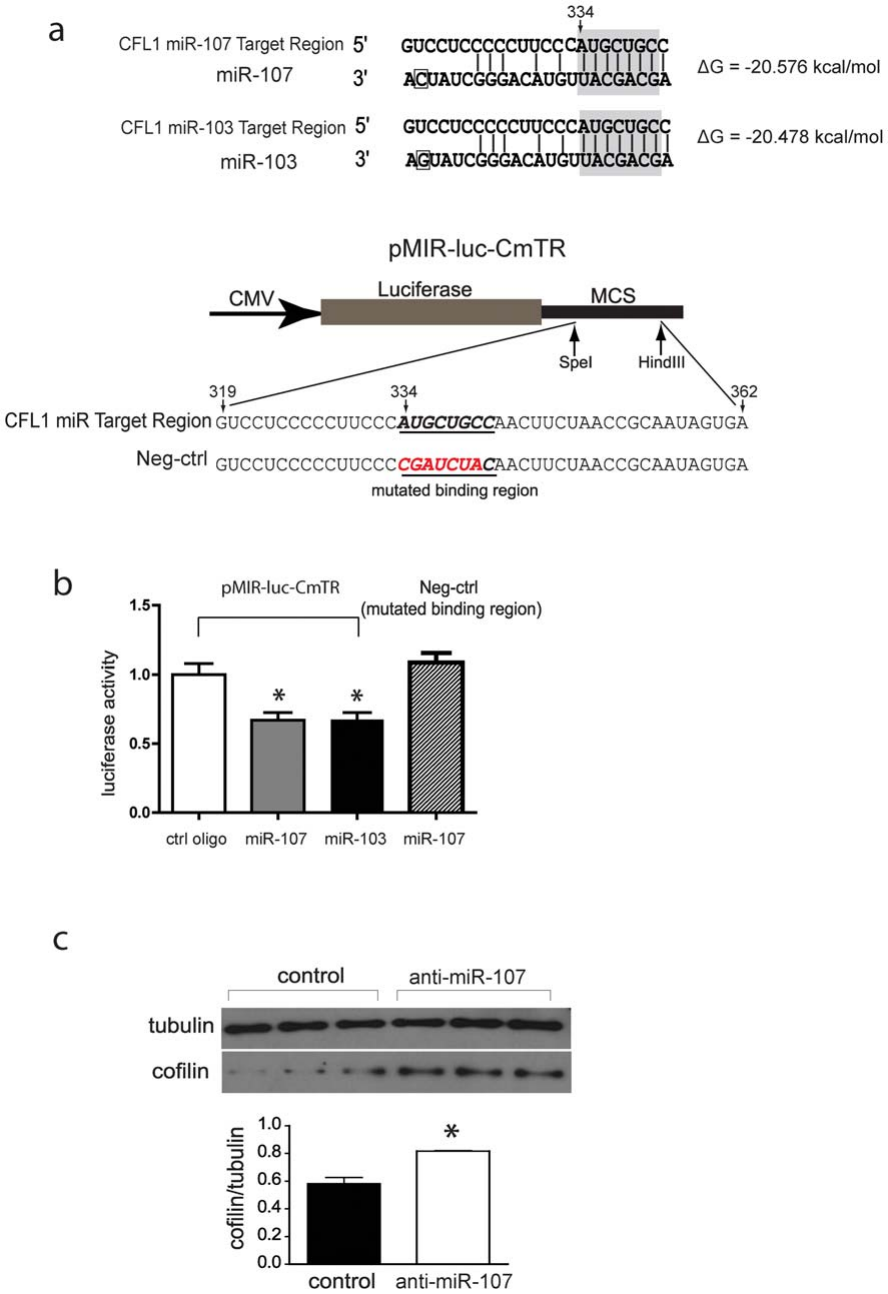
MiR-103 and miR-107 repress cofilin translation. (**a**) Upper panel: MiR-103 and miR-107 are predicted to bind the 3′UTR of cofilin mRNA. Mature miR-103 and miR-107 differ only at the boxed position. The seed region predicted to bind the cofilin 3′UTR is shaded in gray. Lower panel, pMIR-luc-CmTR construct: A portion of the *C*ofilin 3′UTR containing the putative *m*iR-103/107 *T*arget *R*egion (italicized) was inserted downstream of a luciferase reporter. The inserted CFL1 sequence is numbered 319–362 according to NCBI accession NM_005507. As a negative control, the putative miRNA binding site was mutated (red) to examine the specificity of interaction with miR-107. (**b**) MiR-103 and miR-107 repress expression of luciferase from the pMIR-luc-CmTR reporter construct in HEK293 cells. MiR-107 *vs* control, *P = 0.027; miR-103 *vs* control, *P = 0.028; n = 3 wells per miR, ANOVA with post-hoc Scheffé testing. Experiments were replicated 3 times. As a negative control, expression of luciferase from the construct with mutated binding region was no longer suppressed by miR-107. (**c**) Silencing of miR-107 elevates cofilin protein levels in Swe-N2a cells. n = 3 plates per condition, *P = 0.034, Student's *t*-test.

A translational regulatory mechanism of current interest involves miRNAs. We hypothesized that miRNAs might be responsible for control of cofilin translation. Sequence analysis predicted that the 3′UTR of cofilin mRNA is targeted by miR-103 and miR-107 (Fig. 4a, upper panel). To confirm a functional interaction between miR-103 or miR-107 and the predicted target region in the cofilin 3′UTR, we constructed a miR luciferase reporter, pMIR-luc-CmTR (***C***ofilin ***m***iRNA-103/107 ***T***arget ***R***egion, Figure 4a, lower panel). We then co-transfected the pMIR-luc-CmTR reporter construct with miR-103, miR-107, or control oligos into HEK293 cells.

At 72 hours, the samples co-transfected with miR-103 or miR-107 had a 33% decrease in luciferase activity compared to samples co-transfected with control oligo (Fig. 4b). These results suggest that miR-103 and miR-107 bind the putative target sequence in the cofilin 3′UTR and functionally repress translation of the upstream gene. As a negative control, the target region putatively binding miR-107 was mutated, and co-transfection with miR-107 no longer repressed luciferase expression (Fig. 4b).

The luciferase experiment used only a fragment of the cofilin 3′UTR, and it is possible that inclusion of the entire 3′UTR might have different results. Thus, as further evidence of a functional interaction between miR-107 and endogenous cofilin mRNA, we determined whether silencing miR-107 would elevate cofilin protein levels. Since mature miR-103 and miR-107 share the same seed region and are otherwise identical except for one nucleotide (Fig. 4a), and since both identically repress expression of the luciferase reporter construct (Fig. 4b), we only tested miR-107. We transfected anti-miR-107 inhibitor or control oligo into Swe-N2a cells. After 72 hours, the cells in which miR-107 was down-regulated had higher cofilin protein levels compared to controls (Fig. 4c).

### Mature miR-103 and miR-107 levels are decreased while cofilin levels are elevated in APP transgenic mouse brains

**Figure 5:**
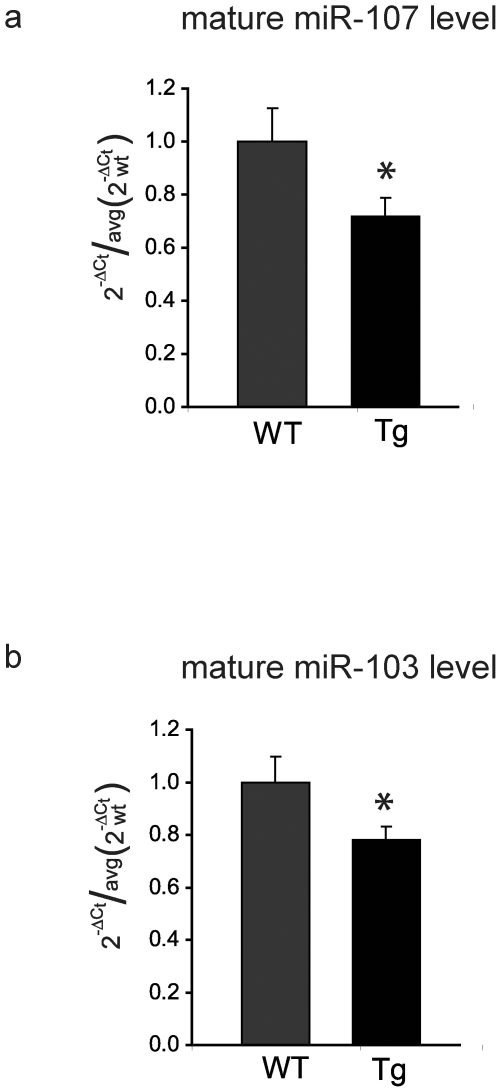
MiR-103 and miR-107 levels are decreased in APP transgenic mouse brains. (**a**) Levels of mature miR-107 are significantly decreased in brains of 4 month-old Tg19959 mice compared to wild-type littermates. N = 5 brains/genotype, each brain in triplicate, *P = 0.04, Student's *t*-test. Cofilin protein levels in these same brains are increased (Figure 1a). (**b**) Levels of mature miR-103 are significantly decreased in brains of 4 month-old Tg19959 mice compared to wild-type littermates. N = 5 brains/genotype, each brain in triplicate, *P = 0.04, Student's *t*-test. Cofilin protein levels in these same brains are increased (Figure 1a).

We next examined mature miR-103 and miR-107 levels in 4-month-old Tg19959 mouse brains by reverse transcription followed by Taqman real time PCR. Brain levels of mature miR-107 were decreased by 28% and miR-103 by 22% in Tg19959 mice compared to wildtype littermates (Fig. 5).

These samples were taken from the same brains in which cofilin protein levels were measured and found to be elevated in Tg19959 mice (Fig. 1a).

### Overexpression of active cofilin is sufficient to induce formation of cofilin rods in primary neurons

**Figure 6:**
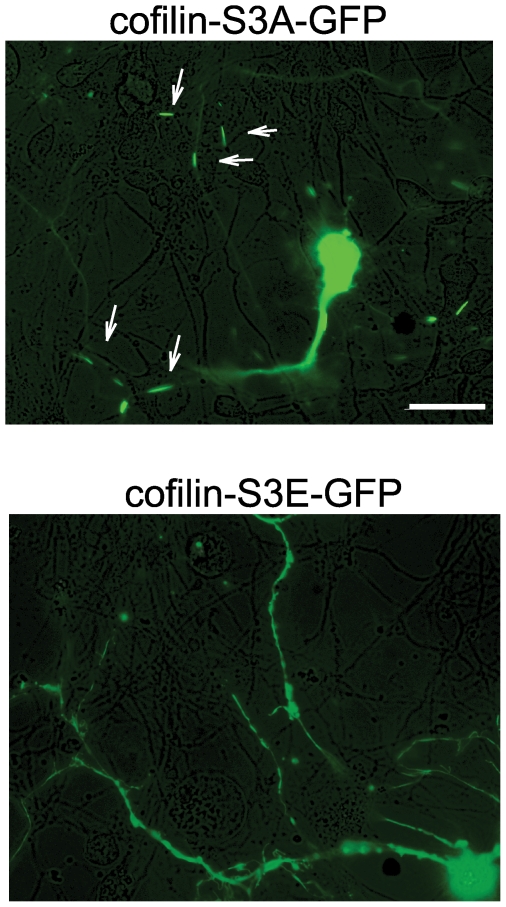
Overexpression of active cofilin (cofilin-S3A, top panel) induced formation of rod-like structures (arrows) in wild-type neurons. The inactive form (cofilin-S3E, bottom panel) did not. Images are merged GFP fluorescence and phase contrast. Scale bar represents 100 µm.

Cofilin protein can be activated through dephosphorylation at residue Ser3 by upstream phosphatases. To determine whether increased expression of active cofilin is sufficient to induce formation of rod-like structures, we transfected DIV6 primary neurons from wildtype mouse embryos with a constitutively active cofilin-GFP construct (cofilin-S3A-GFP). Twenty-four hours after transfection, we observed formation of rod-like structures (Fig. 6, upper panel). As a control, no rod-like structures were observed after transfection with an inactive cofilin-GFP construct (cofilin-S3E-GFP) (Fig. 6, lower panel).

## Discussion

Aggregates of cofilin and actin occur in human AD brains [1], [2], but are less well appreciated than amyloid plaques or neurofibrillary tangles. Rod-like structures containing cofilin and actin also form in cell [3] and animal [9], [18] models of AD. Several lines of evidence suggest that such cofilin-actin rods are pathophysiologically important. First, they form under pathologic conditions, such as energy depletion, excitotoxicity, oxidative stress, and Aβ exposure [3], [7], [8]. Second, they have functional consequences. Feaney and colleagues showed that neurodegeneration in Drosophila and mouse models of tauopathy was associated with formation of actin-rich rod-like structures, and neurodegeneration could be markedly attenuated by genetic manipulations to reduce actin accumulation [18]. Formation of cofilin-actin rods may also be important in recruitment of phosphorylated tau into neuropil threads [9].

Our study investigates the mechanisms underlying the formation of cofilin rods. We show that miR-103 and miR-107 bind to the 3′UTR of cofilin mRNA as predicted, and repress translation of cofilin. Decreasing miR-107 elevates cofilin protein levels, and overexpression of active cofilin induces formation of cofilin-actin rods. Finally, we show in a transgenic APP mouse model that brain levels of miR-103 and miR-107 are decreased, with corresponding increases in brain cofilin protein levels, and formation of cofilin-actin rods or aggregates in primary neurons and brain sections. Since overexpression of inactive cofilin does not induce rod formation, the increased cofilin protein levels seen with decreased miR-103/107 in AD must be followed by activation of the cofilin, in order to lead to rod formation. The regulation of cofilin activity in AD is thus a key topic for future elucidation.

Of note, a recent report suggests that many mammalian miRNAs may work primarily by decreasing levels of target mRNAs ([19] and references therein). Whether this applies in particular to miR-107 and miR-103 and their interaction with cofilin mRNA remains to be elucidated. We did not observe any difference between APP mice and wild type littermates in cofilin mRNA levels, suggesting miR-103 or miR-107 may regulate cofilin expression primarily by decreasing translational efficiency.

These are the first observations to indicate that miRNA involved protein regulation may contribute to formation of abnormal cytoskeletal aggregates in AD pathogenesis. Of note, miR-107 levels are also decreased in human AD brain, where it was associated with increased expression of BACE1 [12]. This previous work connects miRNA with amyloid accumulation. However, it is well-established that a given miRNA may regulate multiple targets [20]. Our data supports an additional role for miR-107 in AD pathogenesis, through upregulation of cofilin and subsequent perturbation of the cytoskeleton.

## Materials and Methods

### Ethics Statement

All experiments were approved by the Institutional Animal Care and Use Committee at Weill-Cornell Medical College. Protocol number: 2010-0025.

### Experimental animals

Tg19959 mice were obtained from Dr. George Carlson (McLaughlin Research Institute, Great Falls, MT). Tg19959 mice were constructed by injecting FVB×129S6 F1 embryos with a cosmid insert containing human APP_695_ with two familial AD mutations (KM670/671NL and V717F), under control of the hamster PrP promoter [21].

### Primary neuronal culture and N2a cell line

Primary neuronal cultures were derived from cerebral cortex and hippocampus of embryonic day 18 Tg19959 or wild-type littermate embryos, as described previously [22] with modifications. Cortices and hippocampi were incubated with 0.25% trypsin and then triturated in glass pipettes. Dissociated neurons were plated initially in 10% FBS/DMEM on poly-D-lysine-treated (0.1 mg/ml; Sigma) coverslips or dishes. They were switched 2 hours later into serum-free Neurobasal media with B27 supplement (Invitrogen, Gaithersburg, MD) and 2 mM L-glutamine. For immunofluorescence, ∼4×10^5^ neurons were plated per well in six-well plates containing coverslips. For western blot, ∼3.3×10^6^ neurons were plated per 100-mm-diameter dish. Genotyping was performed on cerebellum from the same embryo.

Mouse N2a neuroblastoma cells stably transfected with human APP695 carrying the 670/671 Swedish mutation (Swe-N2a) were grown as described previously [23].

### Sample preparation from brains and cells

Mice were deeply anesthetized with intraperitoneal sodium pentobarbital and transcardially perfused with ice-cold saline. The brains were removed and dissected on ice. One hemisphere was stored in RNA Later Solution (Ambion) for subsequent Trizol RNA extraction (Invitrogen). The frontal third of the other hemisphere was homogenized in RIPA buffer for western blot analysis. Primary neurons and N2a cells were homogenized and prepared in Trizol for RNA extraction and in RIPA buffer for protein extraction. Protein concentrations were determined by BCA protein assay (Thermo Scientific).

### Western blot analysis

Samples with equal protein amounts were separated by Tricine-SDS gel electrophoresis and transferred to PVDF membrane using the iBlot dry blotting system (Invitrogen). Membranes were blocked with 5% milk/0.1% Tween20 in TBS for 1 hour at room temperature, followed by incubation with primary antibodies overnight at 4°C. Signal was detected using HRP-conjugated secondary antibodies and enhanced chemiluminescence (Thermo Scientific). Blots were scanned at 300 dpi and densitometry was performed using ImageJ 1.42q (NIH). Antibodies were mouse monoclonal anti-tubulin (Sigma, 1∶10000), rabbit polyclonal anti-cofilin (Cytoskeleton Inc., 1∶500), HRP-conjugated goat anti-mouse IgG (1∶2000) and goat anti-rabbit IgG (1∶3000) (KPL).

### Fluorescence immunostaining, microscopy, and quantification

Primary neurons were fixed with 4% paraformaldehyde + 0.1% glutaraldehyde + 1% sucrose in PBS and permeabilized with ice cold methanol at −20°C for 3 minutes. They were blocked with 2% normal goat serum + 2% BSA in TBS [3], followed by incubation with anti-cofilin antibody (rabbit polyclonal, Cytoskeleton Inc., 1∶500), anti-actin antibody (mouse clone AC-40, Sigma, 1∶100), and then Alexa 488-conjugated donkey anti-rabbit IgG and Alexa 546-conjugated goat anti-mouse IgG (Invitrogen). For rhodamine-phalloidin staining, cells were permeabilized with Triton X-100 at room temperature for 10 minutes instead of ice-cold methanol [3]. Brain slices were fixed in 4% paraformaldehye and permeabilized with ice cold methanol at −20°C for 5 minutes and then processed as above for cofilin.

Immunofluorescence was examined by confocal microscopy using an Axiovert 100M inverted microscope equipped with an LSM 510 laser scanning unit and a 63×1.4 NA plan apochromat objective (Zeiss), Ar488, HeNe1543 nm lasers, and LP560 and BP505–530. Optical sections were no thicker than 0.8 µm. Pseudo color was added to images from different channels using Adobe Photoshop. Cofilin rods were identified as thread-like structures densely immunoreactive to cofilin antibody along neurites. To compare the frequency of rods in primary neurons from transgenic mice and wild-type littermates, at least two hundred neurons were examined for each genotype, and the fraction of neurons with rods was quantified, as previously described [3].

### RT-PCR of cofilin mRNA

Total RNA was extracted from fresh 4-month-old mouse brains using Trizol (Invitrogen) standard extraction. cDNA synthesis and PCR analysis were performed using the cDNA reverse transcription kit (Invitrogen) and SYBR Green PCR Master Mix (Applied Biosystems). Hprt1 was utilized as internal control. In preliminary studies, amplification curves for Hprt1, normalized to the same amount of RNA template, were identical between Tg19959 and wildtype littermate mice. Hprt1 has also previously been used as a housekeeping control in AD [24].

### Bioinformatic Predictions

The cofilin 1 (CFL1, non-muscle) mRNA sequence (NCBI accession NM_005507) was obtained from NCBI. Potential binding between cofilin mRNA and miRNAs was analyzed using the online resources at microrna.org, pictar.org, targetscan.org, microrna.sanger.ac.uk, and sfold.wadsworth.org. MiR-103/107 binding to CFL1 is listed as “target sites of conserved microRNA with good mirSVR scores” at microRNA.org.

### Plasmids

#### pMIR-luc-CmTR

A 44-bp fragment of the ***C***ofilin 3′UTR containing the potential ***m***iR-103/107 ***T***arget ***R***egion was synthesized (Invitrogen) and inserted into the multiple cloning sequence of pMIR-Report (Ambion), downstream of luciferase (Fig. 4a, lower panel). As a negative control, a fragment in which the putative miRNA binding site was mutated at all 7 residues was inserted into the same pMIR-Report plasmid. The 7 mutated nucleotides of the inserted CFL1 mRNA fragment were at positions 334–340 (from the 5′ end of NCBI accession NM_005507), corresponding to positions 2–8 from the 5′ end of miR-107/103 (the “seed region”). **Cofilin-S3A-GFP and cofilin-S3E-GFP** plasmids were generous gifts from J. Bamburg (Colorado State University, Fort Collins, CO). The human cofilin S3A (constitutively active form) and S3E (constitutively inactive form) were inserted into pEGFP-N1, generating a fusion fluorescent protein to C-terminal of cofilin S3A and S3E [3], [7]. Cultured wildtype primary neurons at DIV6 were transfected using lipofectamine 2000 (Invitrogen), according to the manufacturer's instructions. Live images were captured 24 hours after transfection by fluorescence microscopy.

### MicroRNA functional assay

HEK293 cells were transfected using lipofectamine 2000 (Invitrogen), according to the manufacturer's instructions. HEK293 cells are standardly used for luciferase assays and do not express miR-103 or miR-107 [25], [26]. Cells were co-transfected with (1) pMIR-luc-CmTR or the negative control construct with mutated binding region, (2) miR-103, miR-107, or control oligos for miRNAs, and (3) a β-galactosidase control vector provided for normalization of transfection efficiency (pMIR-REPORT miRNA Expression Reporter Vector System, Ambion). In preliminary studies, samples were analyzed at 24, 48, and 72 hours. The greatest changes occurred at 72 hours, which was the time point subsequently used. Luciferase and β-galactosidase activities were measured using the Dual-Light System (Applied Biosystems) and an Optocomp I luminometer (MGM Instruments) according to the manufacturers' instructions. For each sample, the luciferase reading was divided by the β-gal reading. All luciferase/β-gal ratios were then divided by the average luciferase/β-gal ratio of the control samples. These normalized ratios are plotted in Fig. 4b.

### MicroRNA silencing

Swe-N2a cells were transfected using lipofectamine 2000 (Invitrogen) with an anti-miR-107 silencing oligo or control oligo (Ambion). Cofilin protein levels were analyzed after 72 hours by western blot as described above.

### Quantitation of endogenous mature miR-103 and miR-107

Total RNA, including small RNAs, was extracted from fresh 4-month-old mouse brains using the mirVana miRNA Isolation Kit (Ambion). Mature miRNA levels were measured by sequence-specific reverse transcription followed by real-time PCR using Taqman probes for detection. Kits containing the RT primers, PCR primers, and Taqman probes were obtained from Applied Biosystems (Taqman microRNA assay kit 4427975, assay ID 000439 for miR-103, assay ID 000443 for miR-107). Let-7b was used as housekeeping internal control (assay ID 000378). There was no statistically significant difference between let-7b levels in human AD and control brains [13].

For each sample, the cycle number C_t_ to reach threshold fluorescence was determined in triplicate for each miRNA and let-7b. ΔC_t_ (miRx)  =  C_t_ (miRx) – C_t_ (let-7b) was calculated, and Student's t-test was performed to compare ΔC_t_ (miRx) in Tg19959 mice vs wildtype littermates. To determine relative amounts of miRx in Tg19959 mice vs wildtype mice, data are presented using the 2^−ΔΔCt^ method. For each sample and miRNA, 2^−ΔCt^ was calculated, and these values were divided by the average 2^−ΔCt^ in wildtype brains. Figure 5 shows 2^−ΔCt (Tg or wt)^/average (2^−ΔCt (wt)^).

*Statistics.* Unpaired comparisons between two groups were performed using Student's *t*-test assuming equal or unequal variances as determined by F-test. Comparisons involving more than two groups were performed using analysis of variance with post-hoc Scheffe testing to adjust for multiple comparisons. The alpha level for all tests was set at 0.05. Calculations were performed using Excel 2003 (Microsoft), Prism 5.01 (Graphpad), or Statview 5.0.1 (SAS Institute). In all figures, bar graphs show mean ± standard error of the mean.

## Supporting Information

Figure S1 **Cofilin rods (top panel) in Tg19959 primary neurons are also recognized by an antibody to total actin (bottom panel).** Neurons were permeabilized with ice cold methanol. Arrows indicate cofilin rods overlapping with actin staining. Scale bar represents 100 µm.
(TIF)
