# Supplementary figures and images for: MicroRNA-Related Cofilin Abnormality in Alzheimer's Disease
Source: PLoS One. 2010 Dec 16;5(12):e15546. doi: 10.1371/journal.pone.0015546 (PMC3002958; doi:10.1371/journal.pone.0015546)

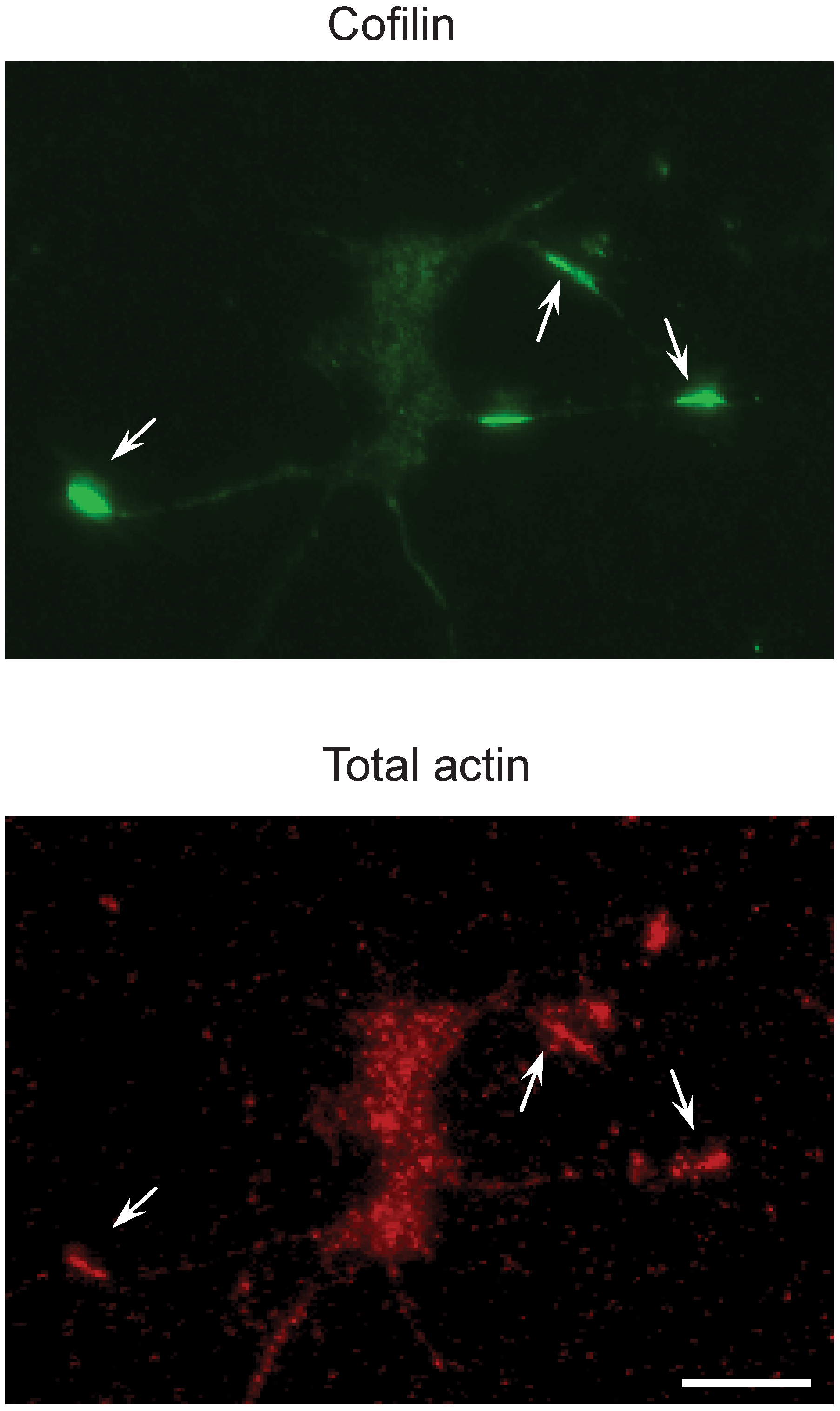

Supplement: Figure S1 — Cofilin rods (top panel) in Tg19959 primary neurons are also recognized by an antibody to total actin (bottom panel). Neurons were permeabilized with ice cold methanol. Arrows indicate cofilin rods overlapping with actin staining. Scale bar represents 100 µm. (TIF) [file pone.0015546.s001.tif]
